# Identification and expression analysis of two steamer-like retrotransposons in the Chilean blue mussel (*Mytilus chilensis*)

**DOI:** 10.1186/s40659-024-00498-x

**Published:** 2024-04-26

**Authors:** Gloria Arriagada, Johan Quezada, Nicolas Merino-Veliz, Fernando Avilés, Diana Tapia-Cammas, Jorge Gomez, Daniela Curotto, Juan A. Valdes, Pablo A. Oyarzún, Cristian Gallardo-Escárate, Michael J. Metzger, Marco Alvarez

**Affiliations:** 1 Instituto de Ciencias Biomédicas, Facultad de Medicina y Facultad de Ciencias de la Vida, Universidad Andres Bello, Santiago, Chile; 2 Departamento de Ciencias Biológicas, Facultad de Ciencias de la Vida, Universidad Andres Bello, Santiago, Chile; 3 Interdisciplinary Center for Aquaculture Research (INCAR), University of Concepción, Concepción, Chile; 4 Centro de Investigación Marina Quintay (CIMARQ), Universidad Andres Bello, Quintay, Chile; 5 Pacific Northwest Research Institute, Seattle, USA

**Keywords:** Bivalvia, Disseminated neoplasia, Smooth-shelled blue mussels, Retrotransposons, Steamer

## Abstract

**Background:**

Disseminated neoplasia (DN) is a proliferative cell disorder of the circulatory system of bivalve mollusks. The disease is transmitted between individuals and can also be induced by external chemical agents such as bromodeoxyuridine. In *Mya arenaria*, we have cloned and characterized an LTR-retrotransposon named Steamer. Steamer mRNA levels and gene copy number correlates with DN and can be used as a marker of the disease. So far, the only mollusk where a retrotransposon expression relates to DN is *Mya arenaria*. On the other hand, it has been reported that the Chilean blue mussel *Mytilus chilensis* can also suffers DN. Our aim was to identify retrotransposons in *Mytilus chilensis* and to study their expression levels in the context of disseminated neoplasia.

**Results:**

Here we show that 7.1% of individuals collected in August 2018, from two farming areas, presents morphological characteristics described in DN. Using Steamer sequence to interrogate the transcriptome of *M. chilensis* we found two putative retrotransposons, named Steamer-like elements (*Mch*SLEs). *Mch*SLEs are present in the genome of *M. chilensis* and *Mch*SLE1 is indeed an LTR-retrotransposon. Neither expression, nor copy number of the reported *Mch*SLEs correlate with DN status but both are expressed at different levels among individual animals. We also report that in cultured *M. chilensis* haemocytes *Mch*SLEs1 expression can be induced by bromodeoxyuridine.

**Conclusions:**

We conclude that SLEs present in *Mytilus chilensis* are differentially expressed among individuals and do not correlate with disseminated neoplasia. Treatment of haemocytes with a stressor like bromodeoxyuridine induces expression of *Mch*SLE1 suggesting that in *Mytilus chilensis* environmental stressors can induce activation of LTR-retrotransposon.

**Supplementary Information:**

The online version contains supplementary material available at 10.1186/s40659-024-00498-x.

## Background

Disseminated neoplasia (DN) is a proliferative cell disorder of the circulating cells of bivalve mollusks that has been reported in at least 15 species of marine bivalves world-wide [1–3]. DNs are characterized by the presence of large rounded or oval cells with a high nucleus: cytoplasm ratio, high frequency of mitotic figures in the connective tissue, sinuses, and organs. These cells also have low adherence an pseudopodia formation in fresh preparations (revised by Carballal et al. [3]). In many bivalve species the disease is progressive, and the replacement of normal cells implies the loss of the normal tissue and organs architecture leading to death.

Elucidating the etiology of DN has been a key issue since its discovery, now we know that in at least eight bivalve species [4–8] the disease is transmitted between individuals through a cancer cell line, this is called bivalve transmissible neoplasia (BTN). This does not exclude the possibility of other factors influencing the development of *de novo* neoplasia in other species. The diagnostics of DNs cannot be performed by external examination of the individuals, but molecular diagnostics tools can be applied thanks to the fact that host and cancer cells have different genomes. Indeed, several qPCR have been developed to diagnose MtrBTN [6, 9, 10], a cancer transmissible cell derived from *Mytilus trossulus*. MtrBTN is a specific BTN infecting various *Mytilus* species, including *M. chilensis*. However, if the DN is not a previously described BTN or a conventional cancer, the diagnostics can only be performed by cytology of hemocytes or by histology [3], which requires an expert eye to correctly classify the animals as healthy or diseased, while also been a time-consuming process that must be perform one animal at the time. Therefore, to define molecular markers for a rapid diagnostic is crucial for large-scale testing of mollusks. In *Mya arenaria*, we have cloned and characterized a retrotransposon named Steamer. Steamer mRNA levels and gene copy number correlates with disease status and can be used as a marker of the disease [11]. Although several Steamer-like elements (SLEs) have been identified in other mollusks [12–14], the only mollusk in which a retrotransposon expression and copy number relate to DN is *M. arenaria*.

Natural populations of the smooth-shelled Chilean blue mussel, *Mytilus chilensis* (Hupé, 1854), are distributed across a wide range along the South American coast [15]. Moreover, it is extensively cultivated, primarily in Southern Chile, with an annual production exceeding 400,000 tons [16]. This makes it a significant resource for global blue mussel production, with around 17% of world production in Chile [17, 18]. However, mussel aquaculture production in Chile is threatened by several microorganisms [19–21], marine pollution [22], the climate variability [23–25] and disseminated neoplasia [26, 27] that can impact the larval settlement and growth of mussel populations. Since farmed mussels are collected from the wild at their juvenile stage, seed production can be reduced by all those factors. Therefore, the diagnostic of diseases that threaten both the native population and the farmed individuals is of utmost importance. We wonder if, similar to *M. arenaria*, *M. chilensis* have retrotransposons in its genome that could be used as markers of DN. Here we identify putative SLEs retrotransposons and determine if their copies and/or expression are correlated with DN status to potentially be used as markers of disease.

## Methods

### Disseminated neoplasia diagnostics

200 individuals of optimal commercial size (∽ 6 cm) were collected during June 2018 from two sites in Chile, Castro (42.4801° S, 73.7624° W) and Calbuco (41.7720° S, 73.1327° W). Hemolymph was drawn as in [28], centrifuged at low speed and the hemocytes were stored in RNAlater (Thermo Fisher Scientific) for RNA and DNA extraction. The animals were then opened and the full body with one valve were fixed for a month with 10% formaldehyde in phosphate buffer pH 6.8. After fixation, 5 mm thick transversal section were cut at 5 mm from the posterior adductor muscle, allowing samples of gills and gonads from the specimens. The tissue was paraffin embedded and then sliced in a Sakura Accu-cut rotative microtome, with R35 low profile blades to obtain a 5 μm histological section, that was deparaffined with xylene and dehydrated with ascendent alcohols. Tissue sections were stained with hematoxylin-eosin and mounted with Entellan mounting medium. The visual analysis of each slide was performed in Olympus CX21LED microscope, along with photographic register using a Nikon 808PureView camera cell phone.

For the diagnosis of the specimens, slides were first pre-screened for the presence of hemocyte infiltration. Then a detailed analysis for selected slides was performed. We observe the hemocyte nuclei characteristics, in search of anomalies as salt and pepper chromatin, size augmentation, irregular nuclear edges and degree of infiltration in gonads and gills tissue was performed. For those animals with infiltration, but no obvious alteration in the hemocytes characteristics, nucleus size was measured in five different field, and use as criteria for final classification. Some of these samples were previously used in the Yonemitsu, 2019 [6] named there Calbuco2, Castro 5, 26, 49, 52 and 84.

### *Mytilus chilensis* transcriptome interrogation and *in silico* analysis

To identify Steamer-like sequences in *M. chilensis* transcriptome, a BlastN search was conducted using as input *M. arenaria* retrotransposon Steamer (KF319019.1) and a transcriptome with the following parameters: E-Value 10^− 3^, word size = 11, Gap cost = Existence 5, Extension 2. The assembly used is deposited into the Sequence Read Archive (SRA) available on the NCBI database, under accession numbers SRX1850489, SRX1850488, SRX1850379, and SRX1847267.

The hits of the transcriptome interrogation (and SLEs identified) were analyzed using Open Reading Frame Finder at National Center for Biotechnology Information (NCBI) [29]. The protein domain search was performed using both Conserved Domain Search Service (CD Search) at NCBI [30] and PROSITE [31]. BlastN was performed against the public sequences of *Mytilus edulis*, *Mytilus galloprovincialis*, and *Mytilus coruscus* found at NCBI. Clustal Omega [32] was used to *Mch*SLEs with a previously described SLE in *M trosulus* (KX018543.1).

### Conventional PCR

For genomic DNA extraction, adductor muscle and mantle of 7 fresh commercially available animals were macerated and digested with 0.1 mg/mL of proteinase K in digestion buffer (100 mM NaCl, 10 mM Tris pH 8.0, 25 mM EDTA, and 0.5% SDS) at 37 °C overnight, after which phenol-chloroform extraction and DNA precipitation were performed. The DNA was resuspended in buffer TE (pH 8.0) and stored at 4 °C. PCR primers were designed to amplify a segment of the reverse transcriptase (RT) domain of *Mytilus chilensis* Steamer-like element 1 (*Mch*SLE1) [SLE1-F (5´cagacgagagcgatagcgaa3´), SLE1-R (5´gcagtcgcaccgctatctaa3´)], or *Mch*SLE2 [SLE2-F (5´tggcgaaggcgatacagaag3´), SLE2-R 5´cgaagtggctcgcaaatgtc3´)]. Primers for α-tubulin (Tub-F 5´gagccgtctgcatgttgagc3´, Tub-R 5´tggacgaaagcacgtttggc3´), a previously described housekeeping gene for *M. chilensis* [33] were used. One hundred ng of gDNA from each animal was used in the PCR assay with GoTaq Flexi G2 (Promega), 2 mM MgCl_2_, 0.5 mM dNTPs, 0.25 µM each primer with a program of 95ºC, 2 min; 35×[95ºC, 30 s; 60ºC, 30 s; 72ºC, 15 s] and a final extension at 72ºC, 5 min. PCR products were visualized on an agarose gel.

### Genome walking

Genome walking to obtain the full sequence of the putative SLEs was performed with DNA isolated from one of the seven animals used in conventional PCR, with the Genome Walker Universal kit (Clontech) according to manufacturer’s instructions. The primers 5′GW-SLE1 (5′gcaagtaagcaatgttgcagtacgtag3′) and 5′GW-SLE1nested (5′gatgcgatttcataattgtcccatgtc3′) were designed for a specific 5′ walk of *Mch*SLE1. For the 3′ genome walk, the primers 3′GW-SLE1 (5′aaatggacgtaccgtaagacgtaacag3′) and 3′GW-SLE1nested (5′cctaaacattcagtcgtgcccgatgttg3′) were designed. The primers 5′GW-SLE2 (5′ccgaagtttgtctggttttacgacag3′) and 5′GW-SLE2nested (5′aacccacggagttggcgttgaaactgg3′) were designed for a specific 5′ walk of *Mch*SLE2. For the 3′ genome walk *Mch*SLE2, the primers 3′GW-SLE2 (5′gccacttcatgctgcaccaaagcgttt3′) and 3′GW-SLE2nested (5′tgcaccaaagcgtttacaacgtatgc3′) were designed. All PCR products were cloned and sequenced.

### SLEs expression and copy number analysis

The hemocytes isolated in RNAlater were collected by centrifugation, RNA and DNA were isolated using TRIzol Reagent (Thermo Fisher Scientific) according to manufacturer´s instructions. cDNA was synthesized using iScript Reverse transcription Supermix for RT-qPCR (Bio-Rad) and 500 ng of RNA. One µl of cDNA was used in the qPCR assay. For genomic DNA the concentrations among samples varied from 2.5 to 470 ng/µl, and up to 100 ng were used in the qPCR assay. The qPCR assay for cDNA and genomic DNA was performed in triplicate for each sample using the α-tubulin, *Mch*SLE1 and *Mch*SLE2 primers described for conventional PCR and the Brilliant II SYBR green qPCR master mix (Agilent technologies) with the normal two step program (15 min, 95 °C; 40 x [10 s, 95 °C; 30 Sect. 60 °C]), with melting curve (1 min, 95 °C; ramp down to 55 °C, ramp up from 55 °C to 95 °C with continued fluorescence collection). The transcript levels and copy number of *Mch*SLEs and α-tubulin was determined using the standard curve method. A plasmid containing a full-length copy of the PCR fragment cloned from a healthy animal of *Mch*SLE1 or *Mch*SLE2 and α-tubulin was used to generate the standard curve. Standard curves based on serial dilutions of the plasmid were created for each qPCR plate and used to calculate the amount of SLE and tubulin. The ratio of SLE and tubulin for each sample were referred to as relative copy number.

From the 200 samples of hemocytes RNA was extracted, 6 had really low amplification, even of the housekeeping gene, thus they were eliminated from the analysis. In the copy number assay, 11 samples did not yield a product in any of the analyzed genes, thus they were excluded from the whole analysis. In 25 of the samples, including two DN, it was not possible to detect *Mch*SLE2, thus they were not considered in the analysis of *Mch*SLE2. Therefore, the analysis only included 194 animals for the RT-qPCR assays, and 175 samples for the copy number detection of *Mch*SLE2.

### BrdU treatment of cultured hemocytes

The in vitro culture of *M. chilensis* hemocytes was perform as reported in [28] with the following modifications: cells isolated from 24 healthy commercial animals were seeded in 24 well plates at a density of 1.5 × 10^4^ cells per well, in triplicate, 24 h later they were treated with 100 mM BrdU for 24 and 48 h or DMSO (vehicle) for 48 h. RNA was extracted using TRIzol Reagent (Thermo Fisher Scientific) according to manufacturer’s instruction and *Mch*SLE1, *Mch*SLE2 and α-tubulin transcripts were detected as described above. For each set of hemocytes, the vehicle treatment was considered as the basal level of expression and arbitrarily set to 1.

### Statistical analysis

All statistical analyses were performed in GraphPad Prism using unpaired t-test 95% confidence or one way ANOVA, a *p* < 0.05 was considered significant. For the BrdU treatment outliers were eliminated before the statistical analysis.

## Results

### Diagnostics of disseminated neoplasia in *Mytilus chilensis*

**Fig. 1:**
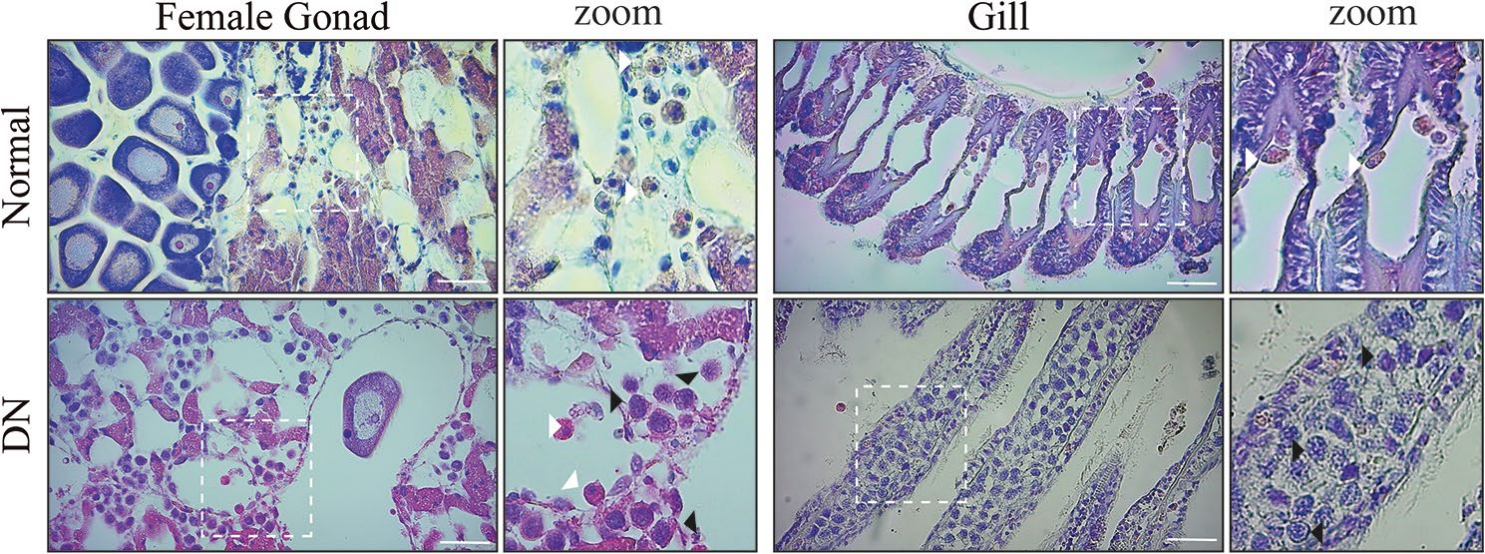
Disseminated neoplasia in *Mytilus chilensis*. To determine if *Mch*SLEs are correlated with the presence of disseminated neoplasia in *M. chilensis*, individuals from 2 different farm sites were diagnosed. Animal were fixed and sliced before hematoxylin-eosin staining. The diagnostic considered level of hemocyte infiltration in the tissue, morphological characteristics, and nuclear size of infiltrating cells. Representative image of female gonad and gills of a normal and a disease animal are shown. A zoom of the area depicted by the withe square is also shwon. Withe arrow heads indicate normal circulating cells, while black arrows indicate cancer cells. DN: disseminated neoplasia. Size bar = 20 μm

Histological analysis was used to diagnose the animals, and samples were considered as positive for DN when hemocyte infiltration, loss of tissue architecture and hemocytes abnormalities were observed (Fig. 1). From our observation 183 animals were considered healthy and 14 as DN (10 in Calbuco, 4 in Castro), three animals from Castro were lost during the histological process. Therefore, from the final 197 animals that could be diagnosed, we established a DN prevalence of 10% in Calbuco and 4.1% in Castro, and a total prevalence of 7.1%, similar to what was reported on a previous study from animals collected in 2016 [26].

Our main question was to know if *M. chilensis* contained in its genome retrotransposons similar to Steamer [12, 13] and test if they correlated with DN as Steamer does [11]. Thus, we saved hemocytes from the diagnosed animals before fix them and in parallel interrogate a transcriptome of *M. chilensis.*

### Identification of steamer-like retrotransposon

**Fig. 2:**
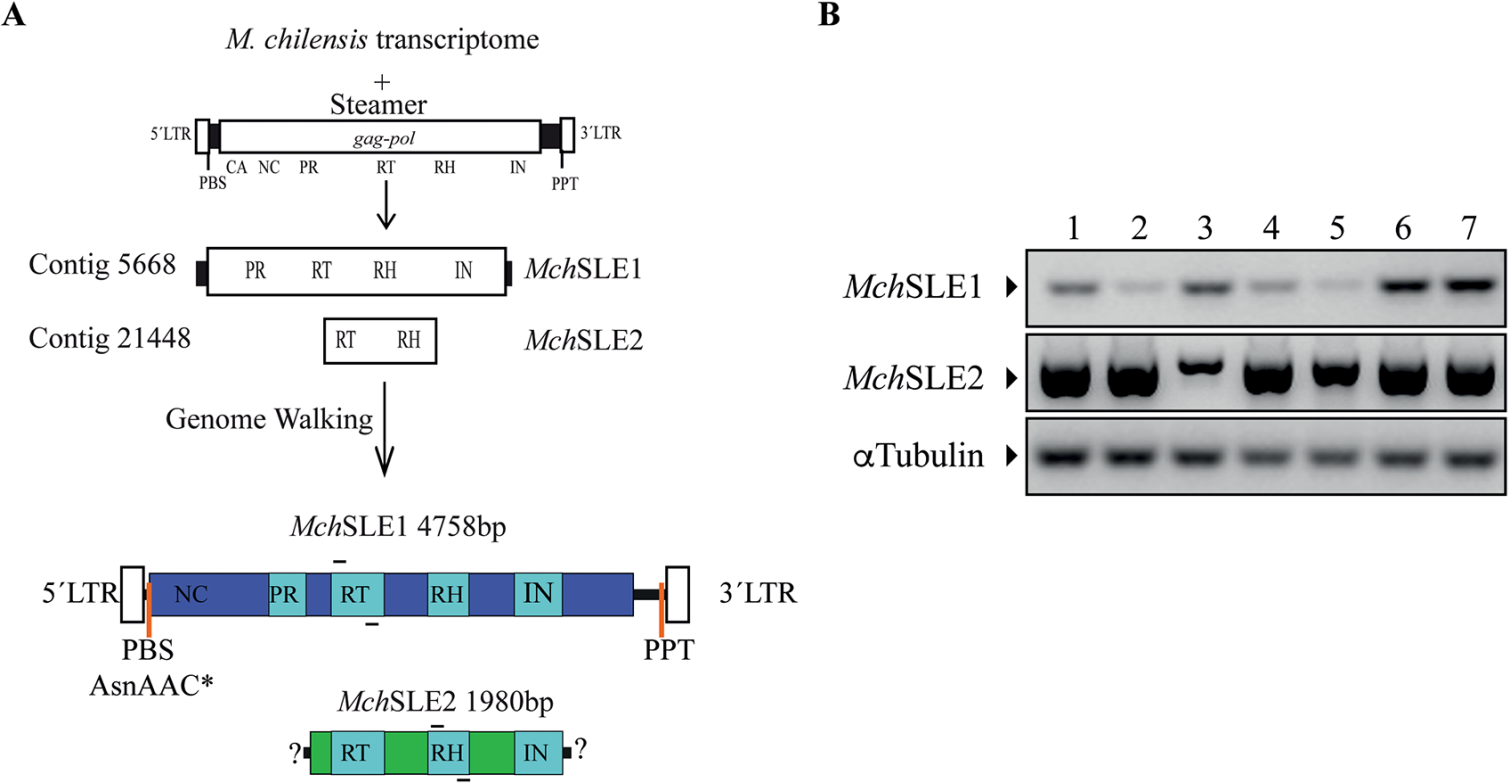
Identification of two Steamer-like elements in *Mytilus chilensis*. **(A)** We interrogate the transcriptome of *M. chilensis* using *Mya arenaria* Steamer retrotransposon, two contig were found and were named *M. chilensis* Steamer-like elements (*Mch*SLE) 1 and 2. Both *Mch*SLEs encode proteins with characteristics of retrotransposons. Genome walking was performed to determine if *Mch*SLEs were indeed LTR-retrotransposons. We identified the full sequence of *Mch*SLE1 and partially extended *Mch*SLE2 up to an integrase domain. *Mch*SLE1 is a 4758 bp LTR-retrotransposons with a single ORF of 4134 bp, long terminal repeats (LTRs) of 185 bp, a putative primer binding site (PBS) for the AsnAACtRNA and a Poly purine track (PPT). **(B)** PCR detection of *Mch*SLE1 (upper panel) and *Mch*SLE2 (middle panel) in the genome of 7 animals, α-Tubulin detection was used as a loading control (bottom panel). The location of the primers used for the PCR assay are depicted above and below the RT domains of both *Mch*SLEs

A raw transcriptome of *M. chilensis* available at NCBI was interrogated using *Mya arenaria* Steamer sequence (KF319019), contigs 5668 (4545 nucleotides) and 21,448 (1158 nucleotides) were selected since both had 64% nucleotide identity to *M. arenaria* Steamer, and upon *in silico* translation both contained proteins with domains of the retroviral Pol protein. They were named *M. chilensis* Steamer-Like Element 1 (*Mch*SLE1) and *Mch*SLE2, respectively (Fig. 2A). *Mch*SLE1 contains a complete open reading frame of 1376 amino acids, with an AUG codon in a Kozak context and a stop codon. *Mch*SLE2 on the other hand, contained a smaller ORF of 385 amino acids, with no obvious initiation or stop codon.

To corroborate if these putative retrotransposons were present in the genome of *M. chilensis*, we obtained animals at the food market, isolated genomic DNA and amplified both, *Mch*SLE1 and *Mch*SLE2 by PCR from each individual. Both putative retrotransposons were detected in the genome of the seven analyzed animals (Fig. 2B).

At the time of this study the genome of *M. chilensis* was not available, therefore we decided to perform genome walking using DNA from commercial healthy animals to extend the sequence of both *Mch*SLEs as we did before for Steamer identification [11]. *Mch*SLE1 contains a single ORF of 4134 bp, flanked by 185 pb direct repeats or LTR (Fig. 2A). The single ORF encode a polyprotein of 1376 amino acids were the most highly conserved corresponds to the retroviral Pol regions, including similarities with the retroviral protease with a DSG active site motif [34]; an RT with a polymerase domain containing an YxDD box [35], RNAse H domain with a diagnostic DEDD catalytic core [36] and an integrase with a HHCC zinc finger and a conserved D, D [35]E motif [37] (Fig. 2A). In the amino terminal of the polyprotein the only Gag similarity is a nucleocapsid domain with two putative zinc fingers containing CCCC and CCHC motif. *Mch*SLE1 contains a putative primer binding site (PBS) for the Asn(AAC) tRNA and a Poly-purine track (PPT) sequence (TGAAAAAAGAAAGGA) located at position 4559. For *Mch*SLE1 genome walking analysis showed us that is indeed a LTR-retrotransposon with a full-length sequence of 4758 bp (OR712106), and strongly suggests that the sequence initially identified in the transcriptome corresponds to the full-length RNA genome of *Mch*SLE1, including the 5´and 3´untranslated regions. *Mch*SLE1 was used later to interrogate the genome of *M. chilensis* and we found 5 copies of this LTR-retrotrasposon distributed in five chromosomes, this was published on [38].

For *Mch*SLE2 we extended the sequence to find a longer ORF encoding protease, RT, RnaseH domains (Fig. 2A) but were not able to find a unique extended sequence further in the 5´or 3´, which suggest this could be the core region of a highly variable mobile element in the genome of *M. chilensis* (OR712107).

### Expression of steamer-like retrotransposon in *M. Chilensis* hemocytes

We have previously shown that Steamer transcripts as well as copy number are correlated with disease status in *M. arenaria* (low in healthy versus high in DN) [11], and Steamer presence can be used as a marker of the disease. Thus, we wondered if *Mch*SLEs were correlated with disease status and potentially be used as a marker of the disease in live animals. To test for expression of *Mch*SLEs RNA transcripts and their copy number, RNA and DNA were extracted from circulating cells of normal and DN individuals and the levels of *Mch*SLE1 and *Mch*SLE2 RNA were determined by quantitative PCR (RT-qPCR) and normalized to a α-tubulin as housekeeping RNA [33].

**Fig. 3:**
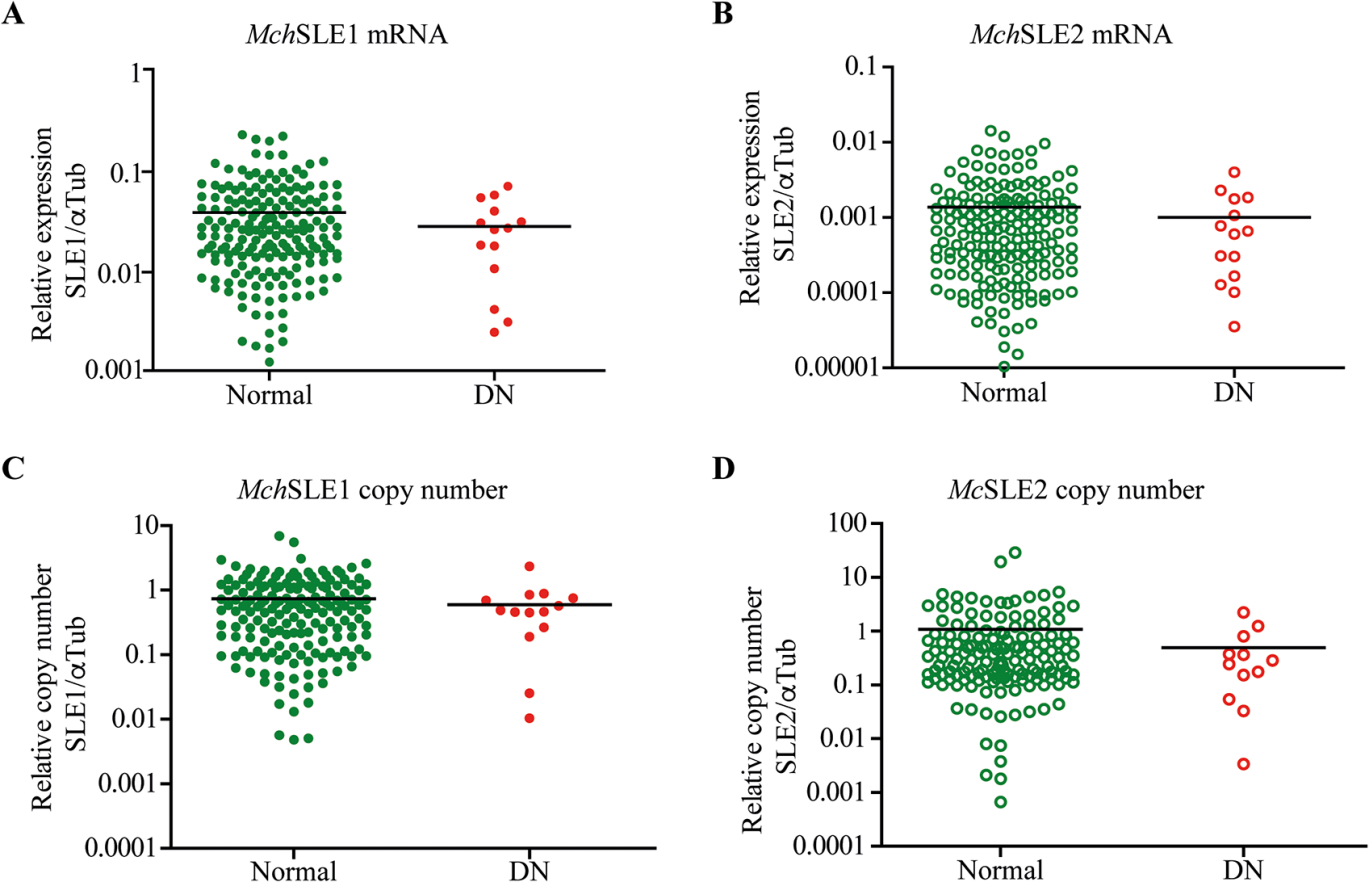
Steamer-like elements present on *M. chilensis* are not correlated with disseminated neoplasia. The expression (**A** and **B**) and copy number (**C** and **D**) of *Mch*SLE1 (**A** and **C**) and *Mch*SLE2 (**B** and **D**) in hemocytes was determined by qPCR. The analysis was performed using the standard curve method, with a plasmid containing one copy of each *Mch*SLE and *Mch*αTubulin amplicon. The expression and copy were normalized to αTubulin. A t-test was performed, and non-significant differences were found among normal and DN animals. Note that Y axis are not on the same scale. The black line indicates the average expression of the animal set

The expression of both *Mch*SLEs was low (compared to tubulin) in normal and DN circulating cells, with no significant differences among them (Fig. 3A and B). Following the same pattern, low relative copy number of *Mch*SLE1 and *Mch*SLE2 were observed among normal and DN animals, and no significant difference was observed between both groups (Fig. 3C and D). These results show that, unlike Steamer, *Mch*SLE1 and *Mch*SLE2 are neither highly expressed, nor expanded in the genome of DN animals, and therefore they cannot be proposed as a molecular marker of the disease.

MtrBTN2 is a cancer transmissible cell derived from *Mytilus trossulus*, that has been previously identified in two of the DN collected samples from this study [6], we therefore compared *Mch*SLEs with an SLE previously described in *M. trossulus* (KX018543.1) and found a nucleotide identity of only a 52.16% and 51.08% for *Mch*SLE1 and 2 respectively (supplementary data 1). Although we did not verify the presence of MtrBTN2 in all the samples diagnosed as affected by DN histology, the facts that the genome of *M. chilensis* showed a single copy of *Mch*SLE1 in five different chromosomes [38], that both *Mch*SLEs can be detected in healthy animal, the high identity with sequences found in the genome of other *Mytilus*, and the fact that no *M. trossulus* genomic or transcriptomic sequence show a hit on our BlastN assays, indicate that *Mch*SLEs do not derive from MtrBTN2, and agrees with the fact that we did not found correlation between copy number and disease status.

### In vitro induction of *Mch*SLEs

**Fig. 4:**
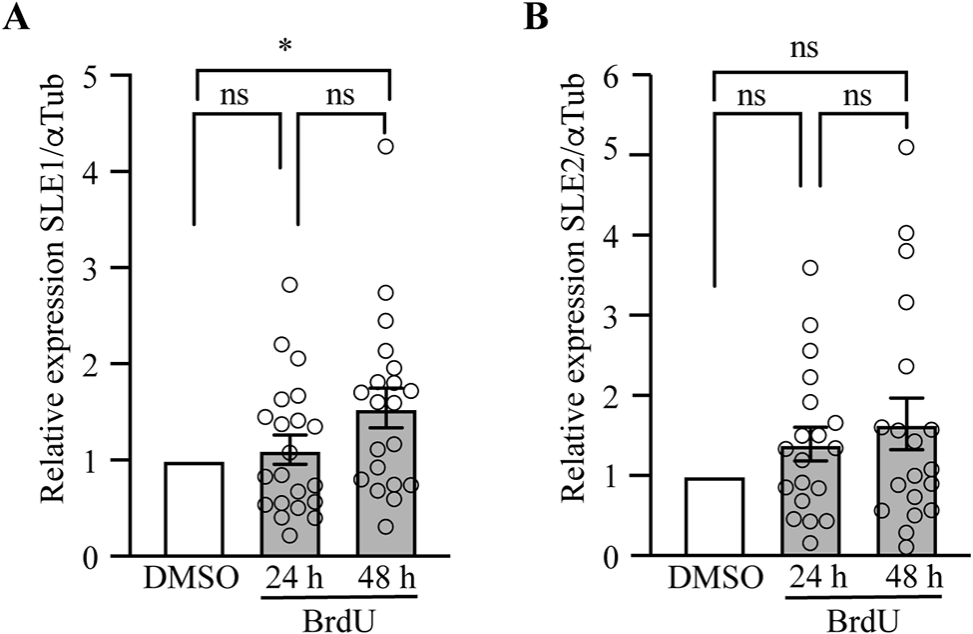
Steamer-like element 1 responds to BrdU treatment. After collection, hemocytes of 20 different animals were seeded in 24 well plate in triplicates and cultured at 10 ºC for 24 h. Then, DMSO or 100mM BrdU was added to each well. Cells were lysed with Trizol at 24–48 h and RNA was extracted to determine the levels of *Mch*SLE1, *Mch*SLE2 and αTubulin transcripts by RT-qPCR. **A**. Relative expression of *Mch*SLE1 upon BrdU treatment. **B.** Relative expression of *Mch*SLE2 upon BrdU treatment. In both, the sum of three experiments performed independently is presented and the DMSO treatment was arbitrary set to 1 for each animal. Please note that Y axis are not in the same scale. One way ANOVA was performed, ns = non-significant, *=*p* < 0.05. Error bars = SEM

Although our results indicate that there is no correlation between expression of *Mch*SLE1 and 2 and DN, there is still expression of these retrotransposons at different levels between individuals. We wondered if as other retrotransposon and endogenous retroviruses, whose transcription is repressed by different epigenetic mechanisms [39, 40] and activated under several stressors [41], *Mch*SLEs transcription could be activated. Bromodeoxiuridine (BrdU) has been shown to be a potent inducer of retrotransposon and endogenous retrovirus transcription [42–44], thus we wondered if in vitro treatment of hemocytes with BrdU could induce or increase the expression of *Mch*SLE1 and *Mch*SLE2. Hemocytes isolated from 24 healthy commercial animals were seeded in 24 well plates and cultured at 10 °C for 24 h, animals were treated with 100 µM BrdU or vehicle, for 24–48 h and the levels of *Mch*SLE1 and *Mch*SLE2 transcripts were analyzed by RT-qPCR. We found that, although for some individuals there is no increase on *Mch*SLE1 expression, the group presents a significantly higher expression at 48 h post-treatment (Fig. 4A), while there are no significant changes in transcription of *Mch*SLE2 (Fig. 4B). This suggests that *Mch*SLE1 transcription can, in some individuals, be induced upon stress as it has been shown for other retrotransposons and for endogenous retroviruses.

## Discussion

Disseminated neoplasia (DN) affects at least 15 species of marine bivalves world-wide [1–3], of those in at least eight bivalve species [4–8] including *M. chilensis*, the disease is also a BTN. The diagnostics of DNs must be performed by direct analysis of hemocytes or the animal tissue [3], requiring an expert eye to correctly classify the animals as healthy or sick. Therefore, to define molecular markers to perform a rapid diagnostic is important to test massively the cultures, especially on farmed mollusks. Here we looked for an LTR-retrotransposon similar to Steamer in the smooth-shelled Chilean blue mussel, *M. chilensis* (Hupé, 1854), that could be use as molecular marker as Steamer can be used for DN in *Mya arenaria* [11]. Although we conclude that SLEs present in the Chilean blue mussel *M. chilensis* are differentially expressed among individuals, SLEs are neither highly expressed, nor expanded in the genome of DN animals. Therefore, they cannot be proposed as a molecular marker of the disease.

Recently, a chromosome level genome assembly of *M. chilensis* was reported [38]. The chromosomal location of the *Mch*SLE1 reported here was analyzed in that report, showing single copy of the full-length retrotransposon in chromosomes 1, 6, 7, 10 and 11. This confirmed that the LTR-retrotransposon *Mch*SLE1 identify here is present in natural population in at least 5 copies. Interestingly the copies present in chromosomes 7 and 11 present a 12 nucleotides insertion in frame with the ORF, that was not cloned in our genome walking analysis. Thus, we cloned *Mch*SLE1 present in either chromosome 1, 6 or 10 and explained the different sequences obtained flanking the LTR in some of the clones amplified in the Genome Walking libraries (data not shown). Because the primers design for qPCR analysis of *Mch*SLE1 copies and expression align on the RT region, further downstream of the 12 nucleotides insertion described in chromosome 7 and 11, we were unable to differentiate which copies were detected in our assays. It could be interesting for future analysis to perform these assays using primer specify for copies present in chromosomes 1, 6, 10 versus 7 and 11, to determine if both are expressed or activated upon a stressor such as BrdU.

We also interrogated the reference genome with *Mch*SLE2 sequence, to our surprise we did not find it in this reference genome. This could mean that *Mch*SLE2 is not really present in the genome of *M. chilensis*, which we do not believe to be true, since we have obtained amplification of this sequence from genomic DNA from more than 200 animals along this study. Most likely this can be explained by the fact that *Mch*SLE2 is not present in the full population of this species, we had animals form Calbuco that did not produce amplification of this sequence from genomic DNA and in a prospective analysis of animals not included in this study, collected from Ancud, only 20% of the animals presented *Mch*SLE2, while all amplified *Mch*SLE1 (data not shown). Further studies are needed to confirm this.

By using degenerate primers in conserved positions in the RT-IN region of the pol *gene* and analysis of the available genomes many SLEs have been identified [12–14], showing that in bivalve species susceptible to DN those retroelements were not amplified [12]. *M. chilensis* was not included on those analysis, therefore it was necessary to test if the *Mch*SLEs found here are amplified in hemocytes derived from DN positive animals. Our results align with the previous reports that shown no amplification of SLEs in DN except for the case of *M. arenaria* [11]. When expression was analyzed, we observed low levels of basal transcription of *Mch*SLEs compared to the α-tubulin housekeeping (Fig. 3A and B), with high variability among individuals, more interestingly we observed that a stressor like BrdU, can increase the levels of both transcripts in some but not all cells isolated from healthy animals in vitro, and a significant higher expression of *Mch*SLE1 is observed at 48 h post BrdU treatment (Fig. 4A). This strongly suggest that at least in *M. chilensis*, LTR retrotransposons expression are responding to environmental stressors. It could be interesting to test if *Mch*SLE1 can respond to natural stressors or harmful substances that can affect natural banks of mollusks. It remains to be tested if this increase in expression involves retrotransposition of *Mch*SLE1, which could lead to genomic instability and oncogenic changes [45, 46], or if leads to adaptation without increasing the copy number. In many bivalve’s species there has been multiple and frequent cross-species transference of SLE elements [13]. When comparing the full length *Mch*SLE1 and Steamer DNA sequences we found a 66.21% of nucleotide identity. We also performed BlastN analysis of *Mch*SLE1 against sequences from other *Mytilus* available at NCBI and founded ten sequences with 100% coverage and 95-99.5% of nucleotide identity derived from *Mytilus edulis*, *Mytilus galloprovincialis*, and *Mytilus coruscus* in agreement to our previous report [38] (Supplementary data 2). *Mch*SLE1could also have been transmitted among those *Mytilus* as reported for other bivalves [13] or could be shared between them from a common ancestor reflecting the complex evolutionary history these bivalves.

The transcriptome analysis and specific strand RT-PCR analysis showed transcripts derived from both strand of *Mch*SLE1 [47], suggesting that both LTRs cand be acting as promoters, our analysis cannot differentiate from which *Mch*SLE1 strand the detected transcripts are being generated, but it will be interesting to test if in any given individual are both transcribed or if they transcribed preferentially. We can speculate that as it has been suggested for retroviruses (Revised in [48, 49]) and Long interspersed nuclear element-1 (LINE-1) [50] the antisense transcripts can be regulators of the sense mRNA, helping to control the expression of the LTR-retrotransposon. Further experiments will be needed to determine if this is indeed happening and if the antisense transcripts or the putative peptides they encode have and any influence on *Mch*SLE1 expression.

Bivalve transmissible neoplasia has been previously shown to affect *M. chilensis* from Argentina and Chile, where MtrBTN2 lineage (derived from *M. trossulus)*, has been previously identify in two DN samples from this study (out of four analyzed DNA isolated from circulation cells) [6]. So far only one SLE has been reported in *M. trossulus* (KX018543.1), but only has 52.16% and 51.08% nucleotide identity with for *Mch*SLE1 and 2 respectively. It would be important for future prospections for DN to look also for MtrBTN2 and these SLEs or others that can be identify as specific of *M. trossulus*, their possible amplification could be used as a marker of the disease.

The impact of DN on mollusk aquaculture and its connection to climate change has not been fully assessed thus far. We are aware that climate change not only directly affects marine environments but can also indirectly contribute to the emergence of additional stressors in ecosystems, including infectious diseases like disseminated neoplasia [51]. Therefore, there is a pressing need to develop efficient diagnostic tools for DN to detect and prevent harmful outbreaks of this disease.

## Conclusions

We conclude that SLEs present in *Mytilus chilensis* are differentially expressed among individuals and do not correlate with disseminated neoplasia. Treatment of hemocytes with a stressor like bromodeoxyuridine induces expression *Mch*SLE1 suggesting that in *Mytilus chilensis* environmental stressors can induce activation of LTR-retrotransposon.

### Electronic supplementary material

Below is the link to the electronic supplementary material.

Supplementary Material 1

Supplementary Material 2

## Data Availability

The data that support the findings of this study are available from the corresponding author upon request.

## Abbreviations

DN: disseminated neoplasia
BTN: Bivalve transmissible neoplasia
BrdU: 5-Bromo-2´-deoxiuridine
SLE: Steamer-like element
ORF: open reading frame
LTR: long terminal repeat
PBS: Primer binding site
PPT: Poly purine track
