# Supplementary material for: Identification and expression analysis of two steamer-like retrotransposons in the Chilean blue mussel (Mytilus chilensis)
Source: Biol Res. 2024 Apr 26;57:17. doi: 10.1186/s40659-024-00498-x (PMC11046912; doi:10.1186/s40659-024-00498-x)
Supplement: Supplementary file 1 — Supplementary Material 1 [file 40659_2024_498_MOESM1_ESM.pdf]

```
CLUSTAL O(1.2.4) multiple sequence alignment


MchSLE1         tgtaacatgtatagtatgtcagtactttgataatgcacgtgtaataactcatgcgcactg     60
KX018543.1      ------------------------------------------------------------     0


MchSLE1         gcacttccacccaactgtgttacacatgttattcttgtaaacaatgtagactaaccatgc     120
KX018543.1      ------------------------------------------------------------     0


MchSLE1         tgtatattaaacgtagttcactataacttggttctctcttaatggtatactatgggatat     180
KX018543.1      ------------------------------------------------------------     0


MchSLE1         aaacatggtgtcagatgttaaaacccgatgtcagacaacgaggaaaacgaaaacataaat     240
KX018543.1      ------------------------------------------------------------     0


MchSLE1         gagccggaagaagagatcatcccagaggaacaagcgatagaaccacaagcagaactagcg     300
KX018543.1      ------------------------------------------------------------     0


MchSLE1         attgaaatggcagccgtcccaccattgtttaacattccattcccggggaaactggacttg     360
KX018543.1      ------------------------------------------------------------     0


MchSLE1         gatggaaatatatcaacaaactggaaaaaattcaagcggacatgggacaattatgaaatc     420
KX018543.1      ------------------------------------------------------------     0


MchSLE1         gcatcaggtttatctacaaaagatacaaaactacgtactgcaacattgcttacttgcatt     480
KX018543.1      ------------------------------------------------------------     0


MchSLE1         ggcccagaagcaatggatatttttgatggactagctttcgaaaatgaagaagacaagaag     540
KX018543.1      ------------------------------------------------------------     0


MchSLE1         gacatagcaaaagtgatcgaaaagtttgaagcgttttgtataggcaaaacaaacgaaaca     600
KX018543.1      ------------------------------------------------------------     0


MchSLE1         tttgaaagatacactttcaacatatgtaatcaaagcgaagccgaaaacatcgacacttac     660
KX018543.1      ------------------------------------------------------------     0


MchSLE1         gtctcaaagctaagaaaacttgcaaaaacatgcaactaccaggatttaatagaaagcctc     720
KX018543.1      ------------------------------------------------------------     0


MchSLE1         atccgtgatagaattgtctgtggaatacgggacaacacggcaagaaaaagacttttgcaa     780
KX018543.1      ------------------------------------------------------------     0


MchSLE1         gaagacaaattgactttgaataagtgcatagatatttgccgtgctttagaatctacttca     840
KX018543.1      ------------------------------------------------------------     0


MchSLE1         gcaaaaatgaaaaccatgacaggagcatctaacaacttggataaaggagaagacattaag     900
KX018543.1      ------------------------------------------------------------     0


MchSLE1         gcggtaagaagtaaatcatcttttaacaagcataaaacatttacacaaaagcaaagcaaa     960
KX018543.1      ------------------------------------------------------------     0


MchSLE1         agaaactgttcctactgtggaaaacaatgcactaaaggaaaatgtcctgcatatggaaaa     1020
KX018543.1      ------------------------------------------------------------     0
```

```
MchSLE1       aagtgcgacagttgcggcaaactaaatcatttcgcttcccaatgcagacaaaaaatgaaa    1080
KX018543.1    ------------------------------------------------------------    0

MchSLE1       cctagaggtaaaagatcagatgtaaggcaatttaacacattatcaacagacgagagcgat    1140
KX018543.1    ------------------------------------------------------------    0

MchSLE1       agcgaatatgaaataatggctgtagaaacagacgatgtaaacgtaaacatgatacagaat    1200
KX018543.1    ------------------------------------------------------------    0

MchSLE1       aagatatttgcaaggatgctattaatagatgagaacaaagaggttaagtttcaattagat    1260
KX018543.1    ------------------------------------------------------------    0

MchSLE1       agcggtgcgactgctaacctaattccgagatcttatctcattcattcacaaattgagagt    1320
KX018543.1    ------------------------------------------------------------    0

MchSLE1       ggaaatgacaagttaacgatgtataatcagtctactatgaaatcgtatggcacatgcatg    1380
KX018543.1    ------------------------------------------------------------    0

MchSLE1       ctgcgtgtaaagaatccaaaaacacacaaacgttacaaggtcaagtttattgttgttgat    1440
KX018543.1    ------------------------------------------------------------    0

MchSLE1       gataaatacacacctctactaggagcaaaggccattcaagctatgaatctaattaagata    1500
KX018543.1    ------------------------------------------------------------    0

MchSLE1       caatttcaaaacattatggtatgcgataaccagaatgcaacaggcattgcgtctatggaa    1560
KX018543.1    ------------------------------------------------------------    0

MchSLE1       agcattatttctgaatactctgacgtatttgagggagaaggaacatttaagggagcctta    1620
KX018543.1    ------------------------------------------------------------    0

MchSLE1       aaattagaaattgatgagtccatacctcctgttaagtctccgttaagacgcatacctatt    1680
KX018543.1    ------------------------------------------------------------    0

MchSLE1       gcacttaagccgaagttaaagacagaacttcaaagactggaaaaactaggagttattaaa    1740
KX018543.1    ------------------------------------------------------------    0

MchSLE1       ccagttgatacaccaacagattgggtatccagccttgtaattgtcaagaaacctagtggg    1800
KX018543.1    ------------------------------------------------------------    0

MchSLE1       aaaataagactatgcattgacccaaagccattaaataaagcattaaagcggtgtcattat    1860
KX018543.1    ------------------------------------------------------------    0

MchSLE1       ccgttgccaattatcgaagatttacttccagaattaagcaaagctaaagtatattccaag    1920
KX018543.1    ------------------------------------------------------------    0

MchSLE1       tgtgatgttaaaaatggattttggcatgtaaacctagcagacgagtcaagctttttaact    1980
KX018543.1    ------------------------------------------------------------    0

MchSLE1       acttttgaaacaccgttcggaagataccgttggacaaagatgccatttggaatttcgcct    2040
KX018543.1    ------------------------------------------------------------    0

MchSLE1       gcccccgaatatttccaacaatttttggaaagagaaattgaaaatctaccgggagtccga    2100
KX018543.1    ------------------------------------------------------------    0
```

```
MchSLE1         accgtagctgatgatattattatttatggtgaaggacagaccattgaaaatgcaacgctc    2160
KX018543.1      ------------------------------------------------------------    0


MchSLE1         gatcacgatagaaaactcaaagcattattggaccgttgccgcgaacgaaatatcaaaata    2220
KX018543.1      ------------------------------------------------------------    0


MchSLE1         aacaaagacaaacttgttctaagagcaacagaaatgccttatattggacacttactgaca    2280
KX018543.1      ------------------------------------------------------------    0


MchSLE1         gcagaaggagttaagccagatcctgaaaagattgctgcgattgtaaatatggaaaaaccg    2340
KX018543.1      ------------------------------------------------------------    0


MchSLE1         acagacgtaaaaggcgtgcagagactattaggtatgattaactacctaacaaaattcctt    2400
KX018543.1      ------------------------------------------------------------    0


MchSLE1         gaaaacttaagtgatatttgcgagccgataagaaaacttacgcacaaagaaaacgcttgg    2460
KX018543.1      ------------------------------------------------------------    0


MchSLE1         aactggacttttgaacacgacaaagcatttgaaatgattaaaaaagcagtgacaaaaaca    2520
KX018543.1      ------------------------------------------------------------    0


MchSLE1         cccgttctcaagtactttgactctaaatccgaaacaacactacaatgtgacgcttcagaa    2580
KX018543.1      ------------------------------------------------------------    0


MchSLE1         aagggactaggcgcttccctaatgcaagacagtcaaccgattgcttacgctagccgtgct    2640
KX018543.1      ------------------------------------------------------------    0


MchSLE1         ttgacgaaaactgaacagaattatgcacaaatagaaaaagaactcttggcagttgttttc    2700
KX018543.1      ------------------------------------------------------------    0


MchSLE1         ggtatggagaaatttcatcaatatacgtacggacgcaaggtgtacgttgaaagtgaccac    2760
KX018543.1      ----------------------------------------------TTGAAAGCGACCAC    14
                                                              ******* ******

MchSLE1         aaaccgctcgaaagtttgtacaaaaaacctttatatcacgctcctaaaaggttacagaga    2820
KX018543.1      AAACCGTTAGTTGCAATCTACGACAAACCGCTTTATCGAGCTACACCACGTTTACAACGC    74
                ****** * *      * *** * *****  * ****  *** *   * * *****  *

MchSLE1         atgtttctcagattacaacgctatgatatcgaactaaagtacaagcagggtaaatatctg    2880
KX018543.1      ATGTTAATGAAACTACAGCGATATGATTTACGGATTGTGTACGTTCCCGGAAAACTTATG    134
                *****  * * * **** ** ****** *     *   ****   *  ** ***  * **

MchSLE1         tacatagccgatacattatccagagcatatttgagaagcaccgatggcaatgaatcaaaa    2940
KX018543.1      TTTATATCCGATGCACTTAGCAGAGCCTATCTACCGGATAGCAATGACAAACTTATTGAT    194
                *  *** ***** ** *   ****** *** *       * * *** ***        *

MchSLE1         acacaagacgaaatactcttggtgcgctccgacatagaagtcgaagttgaacagataaat    3000
KX018543.1      GATGAACTTGATAT-----TAGTTA-CATTGAAAAACAACTACCTATTTCGTCTATAAAG    248
                    **   ** **     * **   *   ** * * ** *     **      *****

MchSLE1         atgaccgactatttggcaatatcggaagaaagaaaaaaactcatagcacagtctactcaa    3060
KX018543.1      A------------------TAGCGGAAATCAAA---------------GACGCGACCGAA    275
                *                  ** *****   * *                *  * **  **

MchSLE1         caagacaaaacactacaacagcttatcaaaactattgaaaatggctggaataattctg--    3118
KX018543.1      GCCGATGAAAATCTGAGAAAGTTAAGTTCCGTCGTTGTGTCAGGATGGCCAAACAGTAAG    335
                   **  ***  **   * ** * *         ***     ** ***   **   *

MchSLE1         ----aacacccgagtgaactagatccattctataatgtacgtgacgaactttctgtacaa    3174
KX018543.1      GAAATGTTACCCGATGACATTCAGTCTTATTGGAATTTCAGGGATGAAATAACTGTGATT    395
```

```
                         **   ***  *  *  * *  *  *** *  * ** *** *  ****

MchSLE1         aacggcattatattcaaaggtgatcgatgtataataccaaatgcaatgagaaacg---aa     3231
KX018543.1      GATGGACTACTTTACAAAAGCCAACGTATATTCATACCTAAATCACTTCAACGAGAGATG     455
                 * **  *  * * **** *  * **     * ***** **  ** *   *   *

MchSLE1         atactgagccagattcatactcatattggcattgaaggatgtttaaaacgtgcacgagaa     3291
KX018543.1      TTAGTGAAATTGCACGAAGCACATCTTGGCATTGTTAAGACAAAACAGCGTGCCCGTGAA     515
                 ** ***    *    *  * *** *********          * * ***** ** ***

MchSLE1         tgcgtatactggcctagaatgaattcagaattgagagactacatcagtaaatgcgatgtg     3351
KX018543.1      ATACTATTCTGGCGTAATATGAACAGTGATATTGAAAACTTCATTAAAAACTGTAGCATA     575
                    *** ***** **  *****    **  *   * *** *** *  ** **     *

MchSLE1         tgccagtcacttgctatgaaacaaccgaaagaaactttaaagtcacacgacgtgccatcg     3411
KX018543.1      TGTAACAAGTTTAGAAAGGCAAACTGTAGAGAACCGCTCAAATCTCATGATATTCCGTCG     635
                **  *     **   * *  * *    * **** *  * ** ** ** **  * ** ***

MchSLE1         agaccgtgggctaagataggaaccgatttattcacactatcgggaaatgactatttgatt     3471
KX018543.1      CGTCCGTGGGCCAAAGTCGGAA--------------------------------------     657
                 * ******** **  * ****

MchSLE1         actgtagattattacagcagtttttttcgaggtggatagactttatgatacttcgtcaaag     3531
KX018543.1      ------------------------------------------------------------     657

MchSLE1         acagtaatcaataaacttaaacaacatttcgctagatggggaatccctgaaacagttatc     3591
KX018543.1      ------------------------------------------------------------     657

MchSLE1         tcagacaatggaccgcaatattcgtccgaacaatttaaacagttcagcaagcaatgggat     3651
KX018543.1      ------------------------------------------------------------     657

MchSLE1         tttcaacataaaacatctagtccgggacatgcacaatctaatgggaaagtcgaaaacgca     3711
KX018543.1      ------------------------------------------------------------     657

MchSLE1         gtgaaaacagcaaaacgcctaatgcgaaaagcgaaaaaatcaaacagtgacccgtacatt     3771
KX018543.1      ------------------------------------------------------------     657

MchSLE1         gcgttactgaattttagaaatacaccacaacaagtgacaaaatacagtccggtacaacaa     3831
KX018543.1      ------------------------------------------------------------     657

MchSLE1         atgatcaacagaaggacacgaacattgctaccaatgaaatcagcattatttgaatcgaaa     3891
KX018543.1      ------------------------------------------------------------     657

MchSLE1         attccagaaaatgtacgaagcaacatcatggataataaaaaacgtcaagaaaaatattac     3951
KX018543.1      ------------------------------------------------------------     657

MchSLE1         aatagatccgcaagagacttaaacgaacttaaacaaggtgaaattgtcagattaacacca     4011
KX018543.1      ------------------------------------------------------------     657

MchSLE1         caagatcgacaaaaagaatgggaaaaaggagtgatcaagaataaaaaatccgagagatcg     4071
KX018543.1      ------------------------------------------------------------     657

MchSLE1         tatgaagtcgaaaaggaaaatggacgtaccgtaagacgtaacagaaaatatttgcgtaga     4131
KX018543.1      ------------------------------------------------------------     657

MchSLE1         actaatgaacaattcaatccgaaatgtcaaacggaagaaattaacattgatccgaatgaa     4191
KX018543.1      ------------------------------------------------------------     657

MchSLE1         caagagatatctcatgatgaaaacaaaaacaatagttcggaccctaaacattcagtcgtg     4251
```

```
KX018543.1       ------------------------------------------------------------     657

MchSLE1          cccgatgttgaagagaaatctcaaccgtgtagccgttatggaagaccaattcagaaacca     4311
KX018543.1       ------------------------------------------------------------     657

MchSLE1          agctggatgaaagattacgatttaaagtgacttttaacggttgaacattgtaaatattag     4371
KX018543.1       ------------------------------------------------------------     657

MchSLE1          ttagagactagaagttaggtttagttaatttagtatttgaactttaaatgacagtgcctt     4431
KX018543.1       ------------------------------------------------------------     657

MchSLE1          agaaaattcatttagattctagaacatatacatggacagttaaatatagcagttaaagaa     4491
KX018543.1       ------------------------------------------------------------     657

MchSLE1          ttttataattttgttcagtaattattttgagtttagattttctgataatttgatataagc     4551
KX018543.1       ------------------------------------------------------------     657

MchSLE1          ttcaagttgaaaaaagaaaggatgtaacatgtatagtatgtcagtactttgataatgcac     4611
KX018543.1       ------------------------------------------------------------     657

MchSLE1          gtgtaataactcatgcgcactggcacttccgcccaactgtgttacacatgttattcttgt     4671
KX018543.1       ------------------------------------------------------------     657

MchSLE1          aaacaatgtagactaaccatgctgtatattaaacgtagttcactataacttggttctcac     4731
KX018543.1       ------------------------------------------------------------     657

MchSLE1          ttaatggtatactatgggatataaaca 4758
KX018543.1       --------------------------- 657


#  Percent Identity  Matrix - created by Clustal2.1
#
#

     1: MchSLE1      100.00   52.16
     2: KX018543.1    52.16  100.00

CLUSTAL O(1.2.4) multiple sequence alignment


MchSLE2          GCACATATATGCATCTATGAAAATAATAGGCAGAATGAGAAAAGCAACTTAACAAGATTT 60
KX018543.1       ------------------------------------------------------------ 0

MchSLE2          CAATTAGACAGTGGAGCAACATGCAATATAATCACTGCAAGAACATTGAAAGAGCTTGAT 120
KX018543.1       ---------------------------------------------TTGAAAGCG------ 9
                                                              ******* *

MchSLE2          ATTAACCAATTACAGAAAACAAGTCAAATACTGACAATGTACAACAATACCACAATAAAA 180
KX018543.1       ----ACCA----C--------AAACCGTTAGTTGCAATCTACGACAAACCGC-------- 45
                     ****    *        *  *   ** *  **** *** ****  *

MchSLE2          CCGATAGGAACATGCATTCTAAAATTGGTGAATCCAAAGAACAATAATAAGTTCAAGGCA 240
KX018543.1       --------------------------------TTTATCGAGCTA---------------- 57
                                                 *  *  ** * *

MchSLE2          GAATTTGTAGTAGTAAAAGATGGCACTTTGACTCCGTTGTTAGGTAGCAAAGCAGTGCAA 300
KX018543.1       ------------------------------------------------------------ 57
```

```
MchSLE2         GCTATGAATTTGGTAACTGTAAATTATGAAAACATTAAAGCAGTGAGACAGGGTGCATTT 360
KX018543.1      ------------------------------------------------------------ 57

MchSLE2         TTTAAACCCCTTTCAAAAGAAACCGTTATGAAGGAATATGCAGACA--TATTTGAAGGGA 418
KX018543.1      ----CACCACGTTTACAA----CGCATGTTAATGAAACTACAGCGATATGATTTACGGAT 109
                     *** * ** * **    *   * * ** ***  * ***  *  *  ** * **

MchSLE2         CTGGTAAATTGCAAGGAAAATATCATTTAGAATTGGACAATACTGCAAATCCTGTAGTAC 478
KX018543.1      TGTGTACGTTCCCGGAAAACTTATGTTT-------------------------------- 137
                   ***  ** *  * *** *    ***

MchSLE2         ATCCGCCAAGAAAAGTACCTGTCGCTATTAAAGAGAAGCTTAAATCCGAATTGGAAAGAC 538
KX018543.1      ------------------------------------------------------------ 137

MchSLE2         TGACAAAGTTAGAAATTATAAAACCAGTTTCAACGCCAACTCCGTGGGTTTCAAGTTTGG 598
KX018543.1      ------------------------------------------------------------ 137

MchSLE2         TTACTGTCGTAAAACCAGACAAACTTCGGATATGCATTGATCCGAAGCATTTGAACCAGC 658
KX018543.1      -------------------------------------ATATCCGATGCACTT-------- 152
                                                       ****** *** **

MchSLE2         AGTTGAAAAGAAGTCATTATCCGCTACCAACTATCGATGATTTACTACCAGAACTGAGTA 718
KX018543.1      ------------------------------------------------------------ 152

MchSLE2         GAGCGAAGGTTTTTAGTGTCGTTGATGCTAAGAATGGATTTTGGCACGTAGAATTGGACG 778
KX018543.1      -------------------------------------------------------AGCAG 157
                                                                        *  *

MchSLE2         AAGAAAGCTCGTTGTTGACAACATTCAACACACCGTTCGGACGATACAGATGGTTGCGAA 838
KX018543.1      AGCCTATCTACCGGAT-------------------------------------------- 173
                *    * **    * *

MchSLE2         TGCCGTTCGGTTTATCTTCTGCACCAGAAGAATATCAAAGAAGGCAAGACCAAACTGTTG 898
KX018543.1      -----------------------------------------AGCAATGACAAACTTATTG 192
                                                         **  * *** **  * ***

MchSLE2         AAGGTTTGCCAGGTGTACGAAGCATAGTAGATGATATTCT-----TATCTATGGCGAAGG 953
KX018543.1      ATGATGAACTTGATATTAGTTACATTGAAAAACAACTACCTATTTCGTCTATAAAGATAG 252
                * * *   *  * * *  *   *** * * *  *  * *        *****   **  *

MchSLE2         CGATACAGAAGAAGAAGCCATTGATGACCACGATAGGAAATTTCGGGCTTTAATGGAACG 1013
KX018543.1      CGGAAATC--AAAGACGCGACC---GAAGCCGATG-AAAATCTGAGAAAGTTAAGTTCCG 306
                **  *      **** ** *     **   ****   **** *  *    * * *   **

MchSLE2         TTGTCGGGAAAGAAACTTAAAACTTAACAAAGAAAAGTTTAAGTTAAAATTGAAGGAAGT 1073
KX018543.1      TCGTTGTG---------------------------------------------------- 314
                * ** * *

MchSLE2         TCGATTTATAGGACATTTAGTAACCAGCGAAGGTTTGAAGCCGGATCCAGAAAAGATTCG 1133
KX018543.1      ------------------------------------------------------------ 314

MchSLE2         AGCCGTAATTGATATGCCGAAACCGACAGATGTTTCCGGAGTAAGAAGAATTATTGGATT 1193
KX018543.1      ----------------------------------TCAGGATGGCCAAACAGTAAGGAAAT 340
                                                  ** ***     **  * **  * * *

MchSLE2         TGTAACATATCTAAGTAAGTTCTTACCAAAACTTAGCGACATTTGCGAGCCACTTCGAAA 1253
KX018543.1      GTTACCCGATGACA---------------------------------------------- 354
                  ** *  **   *

MchSLE2         GCTAACTTTGAAGGACTCCGAATTTTGTTGGATTGAAAATCATGATAAAGCACTTGAAGA 1313
KX018543.1      ------------------------------------------------------------ 354

MchSLE2         AATCAAAAGGCTAGTGACTGCTGAGCCAGTTTTAAAATATTATGATCCTAAACTACAGTT 1373
KX018543.1      ------------------------------------------------------------ 354
```

```
MchSLE2         AACAGTTCAAAGCGATGCTTCACAAACTGGACTTGGTGCTGCAGTTATGCAAGAAGACAG 1433
KX018543.1      ------------------------------------------------------------ 354


MchSLE2         ACCAGTTGCATATGCGAGTAGAGCTCTCACTGATACCGAAACAAGGTATGCTCAGATAGA 1493
KX018543.1      ------------------------------------------------------------ 354


MchSLE2         GAAGGAACTATTATCAGTCATTTTCGGACTTGAAAAATTTCACTCGTATACATATGGTAG 1553
KX018543.1      ---TTCAGTCTTATTGGAATTTCAGGGATGAAATAACTGTGATTGA-TGGACTACTTTAC 410
                      * * ****  *   **   ***    * ** * * * *        **   **

MchSLE2         AACAGTAAATGTAATATCTGATCATAAACCTCTCGAATCAATCATGAAAAAGCCACTTCA 1613
KX018543.1      AAAAGCCAACGTATATTC-ATACCTAAATCACTTCAACGAGAGATGTTAGTGAAATTGCA 469
                ** **  ** ***   **    * **** * **  **  *   ***  *  *  * * **

MchSLE2         TGCTGCACCAAAGCGTTTACAACGTATGCTTTTACGTTTACAAAAGTACGACATAATCCT 1673
KX018543.1      CGAAGCAC-----------------ATCTTGGCATTGTTAAGACAAAACAGCGTGCCCGT 512
                 *  ****                 **  *   *   ***  * *  **  * *   * *

MchSLE2         GCAATACAGACCAGGAAAGGAGATGTACTTAGCCGATACTTTAAGTAGATCGTACTTAAA 1733
KX018543.1      GAAATACTAT-------------------------------------------------- 522
                * *****

MchSLE2         AGAGACTTCAGCTACAAGTAGCACAGCAGACGAAATCGAAAGTATCAACATGATAGATGA 1793
KX018543.1      ------------------------------------------------------------ 522


MchSLE2         GTTGCCGATATCTGAAGAGCGAATTTCTGAACTTCAAGAACATACTAGGAATGATCAACA 1853
KX018543.1      TCTGGCGTAATATGAACAGTGATATTGAAAACTTCATTAAAAACTGTAGCA--------- 573
                  ** **  ** **** ** **  **   *******  ** *      * *

MchSLE2         AATGCAAGAACTAAAAGAAGTTATCCAGGAAGGATGGTCGAGTAACAAATGGAATGTACC 1913
KX018543.1      ---------TATGTAACAAGTTTAGAAAGGCAAACT---------------GTAGAGAAC 609
                           *  ** *****    * *    *                 * *   * *

MchSLE2         ATCGCATGTATCAATATATTTCGACATCAGAGATGAACTTACATTACAGAATGGTCTTTT 1973
KX018543.1      CGCTCAAATCTCATGATATTCCGTCGCGTCCGTGGG------------------------ 645
                  * **  * ***  ***** ** *      *  *

MchSLE2         ATTCAAAGGAGAGAGTTATTATTCCTAA    2001
KX018543.1      --CCAAAGTCGGAA--------------    657
                   *****  *  *
 Percent Identity  Matrix - created by Clustal2.1
#
#

     1: MchSLE2      100.00   51.08
     2: KX018543.1    51.08  100.00
```
